# Pediatric Extramedullary Epidural Spinal Teratomas: A Case Report and Review of the Literature

**DOI:** 10.1155/2021/6702972

**Published:** 2021-10-07

**Authors:** David G. Deckey, Andrea Fernandez, Nina J. Lara, Steve Taylor, Jamal McClendon, David M. Bennett

**Affiliations:** ^1^Mayo Clinic Arizona, Department of Orthopaedic Surgery, Phoenix, AZ, USA; ^2^University of California Davis, Orthopedic Surgery, Davis, CA, USA; ^3^OrthoArizona, Division of Spine Surgery, Phoenix, AZ, USA; ^4^Phoenix Children's Hospital, Department of Pathology and Laboratory Medicine, Phoenix, AZ, USA; ^5^Mayo Clinic Arizona, Department of Neurosurgery, Phoenix, AZ, USA; ^6^Phoenix Children's Hospital, Department of Neurosurgery, Phoenix, AZ, USA; ^7^Phoenix Children's Hospital, Department of Orthopaedic Surgery, Phoenix, AZ, USA

## Abstract

**Background:**

Teratomas in the pediatric population are most commonly found in the sacrococcygeal region. Pediatric intraspinal teratomas, however, are an exceedingly rare central nervous system (CNS) neoplasm. The clinical presentation of these intraspinal neoplasms can vary significantly and thus can be difficult to identify in infants less than one year of age where verbal expression and motor development are still lacking. *Case Description*. A 7-month-old, previously healthy male presented with a thoracic scoliosis and an asymptomatic right midupper thoracic spinal prominence present since birth. MRI revealed an extensive heterogenous mass in the right epidural space from T5-T6 and the right paravertebral space, resulting in severe spinal stenosis. *Outcome*. Complete resection of the tumor, including a three-level neurotomy, was achieved by posterior decompression/laminectomy. The final tumor was consistent with a mature teratoma. The surgical resection was performed without any immediate complications.

**Conclusions:**

Extramedullary epidural teratomas are exceptionally rare tumors in the pediatric population. Clinical presentation can be ambiguous, particularly in an infant. MRI was useful in suggesting a teratoma as a potential diagnosis and for postoperative surveillance for recurrence. However, histopathological analysis remains the gold standard for definitive diagnosis. Surgical resection is the mainstay of treatment, especially in the setting of cord compression and progressive loss of motor function. Close follow-up is crucial to monitor for progressive spinal deformity or recurrence.

## 1. Introduction

Neoplasms of the central nervous system (CNS) are the second most common malignancy in the pediatric population [1]. However, those occurring within the spinal cord are an exceedingly rare diagnosis. About 1% to 10% of CNS neoplasms in the pediatric population are intraspinal, the most common of which are intramedullary [1–3].

Of the various histological subtypes comprising spinal cord neoplasms, spinal teratomas account for a relatively small percentage. In general, teratomas are germ cell tumors that are an amalgamation of highly differentiated, mature tissues—these frequently contain all three germ cell layers [4]. Most commonly, teratomas in the pediatric population are found in the sacrococcygeal region (41%), ovaries (28%), and testes (7%), though there have been case reports of retroperitoneal teratomas [5, 6]. While most will present as mature teratomas, immature, spinal epidural teratomas have been described in the literature, and demonstrate more aggressive behavior [7]. The overall incidence of intraspinal teratomas is estimated to be between 0.15% and 0.18% [8]. However, these tumors are thought to occur more frequently in children and infants compared to adults, at about 5% to 10% of spinal tumors [9, 10].

Intraspinal teratomas can be found in both extramedullary and intramedullary locations of the spinal cord. Various studies have described intramedullary and extramedullary intradural teratomas in the pediatric population. However, fewer studies exist examining spinal teratomas in epidural locations [11]. Here, we describe a rare case of a large extramedullary teratoma extending from the intradural to the epidural space in a 7-month-old male. The patient's family was informed that data concerning the case would be submitted for publication, and they provided consent.

## 2. Case Description

A 7-month-old, previously healthy male was referred for an initial consultation and evaluation to the neurosurgery clinic of a large children's hospital for a congenital deformity of the spine and an asymptomatic right midupper thoracic spinal prominence present since birth. All subsequent care, including imaging, biopsy, histology, and surgery, was performed at the same institution. He was delivered at term via cesarean section secondary to breech presentation. The patient had met all developmental milestones with no noticeable complaints of pain or neurological deficits.

His neurological exam revealed no motor or sensory deficits in the upper or lower extremities bilaterally. Cranial nerve testing, muscle tone, and deep tendon reflexes were intact. Visual inspection was significant for slight curvature of the spine with no spinal dysraphism or midline stigmata.

**Figure 1:**
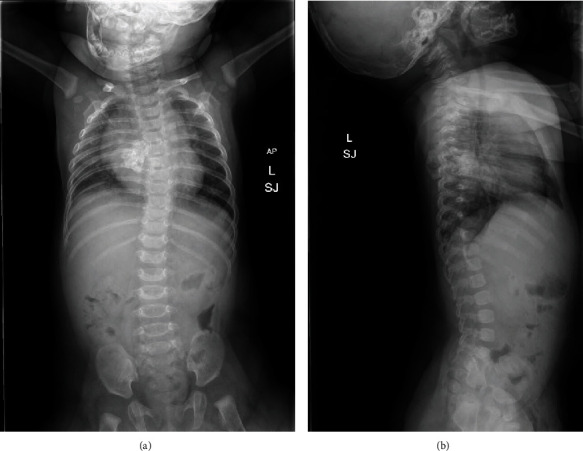
(a) AP spine radiograph showing thoracic levoscoliosis with a calcified lesion projecting from the right posterior aspect of the midthoracic rib segments extending into the soft tissues. (b) Sagittal spine radiograph further demonstrating osseous projection from the midthoracic posterior rib segments.

Imaging was subsequently performed to evaluate the spinal prominence noted on physical exam. Plain films of the thoracic spine demonstrated levoscoliosis and an additional osseous projection from the right posterior aspect of the midthoracic rib segment (Figure 1).

**Figure 2:**
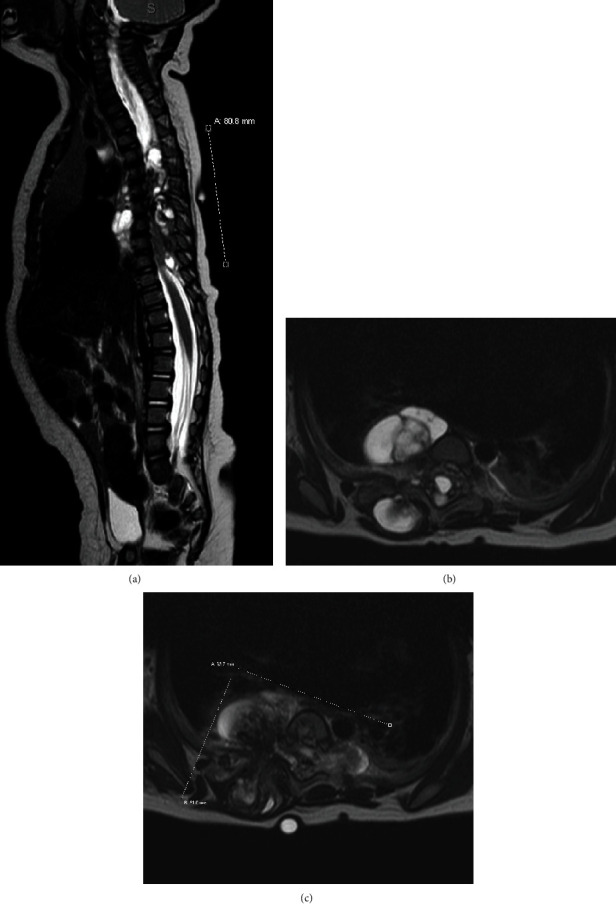
(a) Sagittal T2-weighted MR image showing multilobulated, partially cystic, trans-spatial heterogenous mass extending cranially to T3 and caudally to T10 with soft tissue extension anteriorly into the dorsal aspect of the intrathoracic cavity. (b, c) Axial T2-weight MR image further demonstrating the mass centered at T5-6 paravertebral space extending anteriorly into the dorsal aspect of the intrathoracic cavity, into the right dorsal paraspinal soft tissue/musculature, and into the spinal canal with severe displacement of the cord and resultant spinal stenosis.

Magnetic resonance imaging (MRI) of the cervical, thoracic, and lumbar spine revealed a multilobulated, partially cystic, calcified, heterogenous mass centered at the right (T5-T6) paravertebral space extending anteriorly into the dorsal aspect of the intrathoracic cavity (Figure 2(a)), right dorsal/posterior paraspinal soft tissues/musculature (Figure 2(b)), and spinal canal, resulting in severe displacement of the spinal cord, spinal stenosis and bilateral foraminal stenosis (Figure 2(c)). No intracranial abnormalities were noted. Differential diagnosis included teratoma and neuroblastoma. An open biopsy of the lesion was performed, and histology of the biopsy was consistent with mature teratoma. Throughout the time it took to schedule and perform the biopsy and receive the results (a period of 8 days from initial presentation), the patient's motor exam progressed to mild hypotonia in his lower extremities. This progression began prior to biopsy and was not believed to be a result of the biopsy. After a thorough discussion with the family, the decision was made to proceed with open tumor excision and resection.

The patient underwent open tumor excision and posterior decompression/laminectomy (T3 to T10) due to tumor magnitude. Neuromonitoring was used throughout the case; baseline motor signals were absent at the beginning of the case. During the procedure, three nerve rootlets (T5, T6, and T7) on the right were found penetrating through the tumor, and a three-level neurotomy was performed. The rest of the tumor was gently teased from the dura. Additionally, there was some retropleural extension into the right chest cavity. This was resected as well. Given the location of the tumor, biopsy results, and clinical picture, goals of surgery were to obtain as close to negative margins as possible. During the procedure, multiple specimens from various sites were sent for frozen histopathological analysis. These all came back consistent with mature teratoma, without any malignant features. As the tumor itself was teased off the dura, pleura, and other surrounding structures, true negative margin surgery would have been associated with significantly more morbidity. Considering benign histology, the decision was made to preserve as much function as possible and to proceed to closely monitor for recurrence post-operatively.

**Figure 3:**
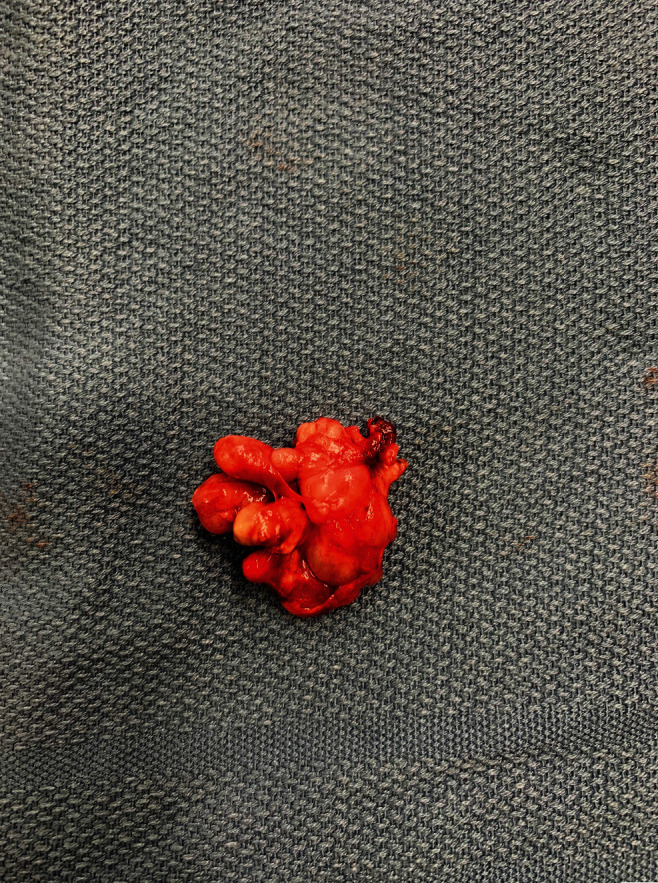
Intraoperative photograph of the resected tumor.

**Figure 4:**
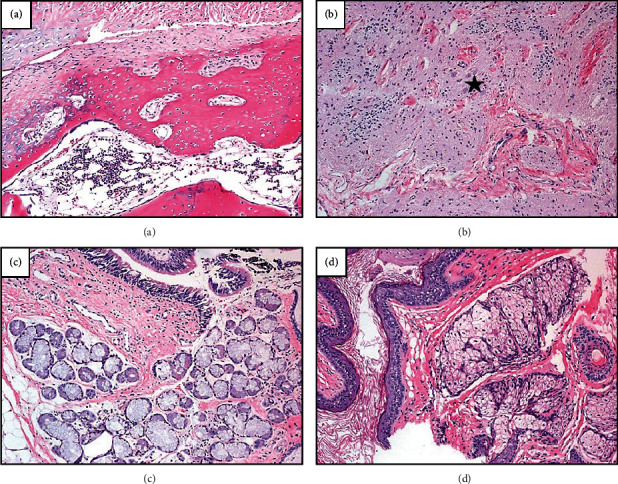
Microscopic review of both excisional biopsy and surgical resection showed similar histologic features comprised of myriad tissue types including bone, cartilage, and marrow elements (a); neuroglial tissue (b) with conspicuous, mature neurons (star), skin with surrounding supporting sebaceous glands (c); and respiratory tract epithelium admixed with serous and mucous glands (d). (a–d) Stained with hematoxylin and eosin and captured at 100x.

Histologically, the tumor revealed skin with adnexal structures and keratinous debris, cystic spaces lined by ciliated and mucinous epithelium, neuroglial tissue, bone with bone marrow, cartilage, and mature fibro-adipose soft tissue. Mature fibrovascular soft tissue, granulation tissue, and a few bone foci were noted at the inked margins of excision. The final pathological diagnosis was consistent with a mature teratoma (Figures 3 and 4).

**Figure 5:**
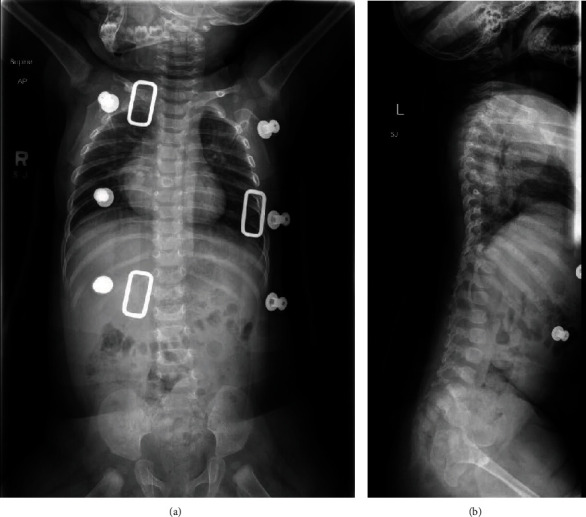
(a, b) Postoperative AP spine and sagittal radiographs with no evidence of residual tumor or further progression of scoliosis.

At the end of the surgical procedure, neuromonitoring reported a partial return of motor activity from a baseline of no activity. The patient tolerated the procedure well and was transferred to the pediatric intensive care unit (PICU) postoperatively for further management. He was transferred to the floor on postoperative day 1 and discharged on postoperative day 2. He returned for a follow-up visit at 1.5 weeks in a thoracic-lumbar-sacral orthosis (TLSO) brace and received full-length plain radiographs (Figure 5). His mother reported improved tone in the lower extremities and ability to stand with assistance.

At his 8-week postoperative visit, he remained in a thoracic lumbar sacral orthosis (TLSO) brace and continued to show improvement in lower extremity strength and ability to maintain posture. At his 6-month and 1-year postoperative visits, the patient was doing well and had progressed to crawling, cruising, and walking. He demonstrated 5/5 strength in both upper and lower extremities throughout all muscle groups. No recurrence of the tumor was noted on repeat plain radiographs, nor at one- or two-year follow-up MRI. At 1- and 2-year follow-up, he had 20-degrees of scoliosis, without any residual neurological deficits. The plan will be for continued bracing, likely until skeletal maturity to slow curve progression. He will continue to have annual follow-up through adolescence to monitor for progressive spinal deformity with plain radiographs, as well as yearly surveillance MRI through 5-year follow-up, and then will decrease frequency to every 2-3 years.

## 3. Discussion

Intraspinal teratomas within the pediatric population represent an exceptionally rare clinical entity. While several case reports and studies documenting intraspinal teratomas have accumulated over time, the large majority of these tumors have been observed in intramedullary or extramedullary intradural locations [11–16]. According to a systematic review of 170 cases conducted by Park et al. comparing the characteristics of spinal intradural teratomas and spinal epidural teratomas, only fourteen cases of spinal epidural teratomas have been reported in the literature to date [11]. Of the total number of cases describing spinal intradural teratomas, 1.9% of those cases occurred in epidural-intradural locations. Ultimately, there is a relative paucity of information regarding clinical characteristics, diagnosis, management, and prognosis of extramedullary epidural teratomas. This report (1) highlights features of an early onset tumor at 7 months of age to aid with diagnosis, (2) helps continue to address the paucity of information on these tumors, in particular the work-up and treatment, (3) shows successful outcome can be obtained even in the setting of a large tumor in a very young child, and (4) outlines surveillance and follow-up.

The clinical presentation of intraspinal tumors can vary significantly and can be difficult to identify in the pediatric population, since complaints are often vague and symptoms nonspecific. Pain is a common presenting symptom of intraspinal tumors, followed by weakness and gait disturbance [1, 3]. However, this constellation of symptoms is not always present and can be difficult to assess, particularly amongst nonambulatory and minimally verbal infants. Thus, other subtler findings on clinical exam are relied upon to strengthen a diagnosis. Clinical features such as progressive scoliosis and anomalies like skin stigmata and spinal dysraphism are shown to occur in conjunction with intraspinal tumors and lesions and can provide important clues to the underlying diagnosis [1, 3, 7, 11]. In the case presented, a thoracic levoscoliosis and spinal prominence were the only significant findings on clinical exam.

Based on heterogenous composition of tissue types within teratomas, several imaging findings are suggestive of a spinal teratoma. MRI of the spine performed in our patient displayed a heterogenous tumor, which was multilobulated, partially cystic and calcified. Despite these findings and advancements in imaging technology, distinguishing between tumor types on imaging studies is unreliable [17, 18]. Histopathological analysis remains the gold standard for identifying the final diagnosis of a teratoma. The clinical utility of imaging studies for intraspinal tumors is in operative planning, including lesion localization, whether it is intra- or extradural and/or intramedullary, and identifying any other associated anomalies, such as split cord malformations, including diastematomyelia and diplomyelia [17, 19].

The primary treatment of intraspinal tumors is surgical resection. In particular, microsurgical dissection, involving laminectomy or laminotomy with tumor resection and nerve root and/or spinal cord decompression, is the standard means for surgical treatment of intraspinal tumors [17]. Depending on the extent of tumor adherence to surrounding structures, subtotal resection has been reported as a means of preserving surrounding tissue while also removing the bulk of tumor. Given the slow growing and benign nature of teratomas, the incidence of recurrence is relatively low, especially if total resection is performed [7]. However, careful monitoring for recurrence remains a potential concern [7, 15, 17, 20]. We recommend annual follow-up with repeat lose-dose, long-standing spine radiographs for progression of scoliosis, as well as yearly surveillance MRIs through 5-year follow-up for disease recurrence. After 5-year follow-up, we plan to decrease frequency of MRI to every 2-3 years, with continued annual radiographic follow-up through skeletal maturity.

Management after resection will involve close clinical and radiologic follow-up and continued bracing, as the authors have concern for the long-term development of a progressive deformity. A recent retrospective review of 272 cases of intracanal tumor resection found the incidence of postoperative progressive spinal deformity to be 15.8% [21]. This occurred mostly in patients who had younger age (≤18 years) or tumors that involved multiple segments and preoperative spinal deformity. As the patient described in this case had all three of these risk factors, the authors will closely monitor for progressive deformity as he ages.

## 4. Conclusion

Extramedullary epidural teratomas are exceptionally rare tumors in the pediatric population. Clinical presentation can be ambiguous, particularly in an infant. MRI was useful in suggesting a teratoma as a potential diagnosis and for postoperative surveillance for recurrence. However, histopathological analysis remains the gold standard for definitive diagnosis. Surgical resection is the mainstay of treatment, especially in the setting of cord compression and progressive loss of motor function. Close follow-up is crucial to monitor for progressive spinal deformity or recurrence.
